# Subjective Birth Experience and Person-Centred Care in Obstetrics: Study Protocol of the Prospective Mixed-Methods Research Project RESPECT

**DOI:** 10.1055/a-2551-3705

**Published:** 2025-06-02

**Authors:** Bianka Vollert, Nina Schurig, Vanessa Zieß, Victoria Weise, Lara Seefeld, Ariane Göbel, Cahit Birdir, Pauline Wimberger, Susan Garthus-Niegel

**Affiliations:** 1 686782 Institute for Systems Medicine (ISM), Faculty of Medicine, MSH Medical School Hamburg, Hamburg, Germany; 2 59988 Institute and Policlinic of Occupational and Social Medicine, TUD Dresden University of Technology, Dresden, Germany; 3 Institute for Medical Information Processing, Biometry, and Epidemiology, Pettenkofer School of Public Health, Ludwig-Maximilians-University Munich, Munich, Germany; 4 59988 Department of Psychotherapy and Psychosomatic Medicine, Faculty of Medicine and University Hospital Carl Gustav Carus, TUD Dresden University of Technology, Dresden, Germany; 5 37734 Department of Child and Adolescent Psychiatry, Psychotherapy and Psychosomatics, University Medical Centre Hamburg-Eppendorf, Hamburg, Germany; 6 Department of Gynecology and Obstetrics, Faculty of Medicine and University Hospital Carl Gustav Carus, TUD Dresden University of Technology, Dresden, Germany; 7 25563 Department of Childhood and Families, Norwegian Institute of Public Health, Oslo, Norway

**Keywords:** subjective birth experience, person-centred care, obstetric violence/mistreatment, parental mental health, mixed-methods, subjektives Geburtserleben, personenzentrierte Versorgung, Gewalt in der Geburtshilfe, elterliche psychische Gesundheit, Mixed-Methods

## Abstract

**Background:**

The interdisciplinary research project **RESPECT** (A Prospective Mixed-Methods- **RE** search Project on **S** ubjective Birth Experience and **PE** rson-centred **C** are in Paren **T** s and Obstetric Health Care Staff) aims to investigate the subjective birth experience of (expectant) parents in Germany and associated factors before, during, and after childbirth, including care-related aspects such as person-centred care and mistreatment during childbirth. In addition, the perspective of obstetric health care staff regarding their role in parents’ subjective birth experience and person-centred care in obstetrics are explored.

**Methods:**

RESPECT _PARENTS_ is a prospective cohort study targeting a community sample of expectant parents in the Dresden area with four assessment points from pregnancy to 24 months postpartum using online questionnaires and a structured telephone interview. Applying a mixed-methods approach, the main study is complemented by a sub-study with parents (RESPECT _PARENTS-TALK_ ) and an additional study branch with obstetric health care staff (RESPECT _STAFF_ ), both using qualitative interviews.

**Results:**

In this study protocol, the theoretical background, methods, and first results regarding sociodemographic and birth-related variables of the sample (N = 2424 participants including n = 1693 expectant mothers/birthing parents and n = 731 partners) are presented and discussed.

**Conclusion:**

The data will provide meaningful insights into parents’ subjective birth experience and health-related effects over time, considering the perspectives of both parents and the obstetric health care staff. The findings can contribute to the development of strategies to improve obstetric health care according to the latest WHO recommendations and the German national health goal “Health around childbirth”, and thus to prevent traumatic birth experiences.

## Zusammenfassung

**Hintergrund:**

Ziel des interdisziplinären Forschungsprojekts **RESPECT** („A Prospective Mixed-Methods- **RE** search Project on **S** ubjective Birth Experience and **PE** rson-centred **C** are in Paren **T** s and Obstetric Health Care Staff“) ist eine gezielte Untersuchung des subjektiven Geburtserlebens (werdender) Eltern in Deutschland sowie der damit in Zusammenhang stehenden Faktoren vor, während und nach der Geburt einschließlich versorgungsbezogener Aspekte wie die personenzentrierte Geburtshilfe sowie Gewalterfahrungen unter der Geburt. Daneben wird auch die Perspektive des geburtshilflichen Personals bezüglich der eigenen Rolle für das subjektive Geburtserleben der Eltern und der personenzentrierten Versorgung in der Geburtshilfe erforscht.

**Methoden:**

Bei RESPECT _PARENTS_ handelt es sich um eine prospektive Kohortenstudie, in der werdende Eltern aus der Allgemeinbevölkerung im Raum Dresden zu 4 Zeitpunkten, beginnend von der Schwangerschaft bis 2 Jahre nach der Geburt, mittels Onlinefragebögen und einem Telefoninterview befragt werden. Entsprechend des Mixed-Methods-Ansatzes wird die Hauptstudie durch eine Teilstudie mit teilnehmenden Eltern (RESPECT _PARENTS-TALK_ ) und einen zusätzlichen Studienzweig mit geburtshilflichem Personal (RESPECT _STAFF_ ) ergänzt, wobei jeweils qualitative Interviews durchgeführt werden.

**Ergebnisse:**

In diesem Studienprotokoll werden der theoretische Hintergrund, die verwendeten Methoden und erste Ergebnisse hinsichtlich soziodemografischer und geburtsbezogener Merkmale der Stichprobe (n = 2424 Teilnehmer*innen, darunter n = 1693 werdende Mütter/gebärende Elternteile und n = 731 Partner*innen) vorgestellt und diskutiert.

**Schlussfolgerung:**

Die Daten werden aufschlussreiche Erkenntnisse über das elterliche subjektive Geburtserleben und gesundheitsbezogene Auswirkungen im Zeitverlauf liefern, während die Perspektiven sowohl der Eltern als auch des geburtshilflichen Personals berücksichtigt werden. Die Ergebnisse sollen zur Entwicklung von Strategien zur Verbesserung der geburtshilflichen Versorgung gemäß den neuesten Empfehlungen der WHO und des nationalen Gesundheitsziels „Gesundheit rund um die Geburt“ in Deutschland beitragen und damit auch traumatischen Geburtserlebnissen vorbeugen.

## Introduction

For both mothers/birthing parents 1 and their partners 2 , the birth of a child is a momentous and transformative event in life. Despite most parents having a positive birth experience, international studies show that up to 44% of mothers and about a quarter of partners report negative or even traumatic birth experiences 1 2 3 4 5 . While negative subjective birth experiences have been the focus of several studies, positive subjective birth experiences have received significantly less attention in research 6 , although both positive and negative experiences may contribute to parents’ postpartum functioning and their family relationships. Furthermore, even though nowadays most partners are present at the birth of their child 7 8 9 , there are few studies that investigate the subjective birth experience of partners and representative results are lacking 10 . However, initial research has shown that a negative subjective birth experience can also have serious implications for the mental health and family relationships of partners 11 12 .

### Relevance of parents’ subjective birth experience

There is a growing body of evidence indicating that parents’ subjective birth experience is of crucial importance for their well-being, functioning, and family relationships in the immediate postpartum period and beyond 3 13 14 . Experiencing childbirth as positive is associated with numerous beneficial outcomes such as enhanced parental mental health and well-being, improved parent-child bonding, initiation and continuation of breastfeeding, and may even predict physical recovery after childbirth 15 16 17 . A positive birth experience can help parents grow into their new role with a high level of self-confidence and personal strength, which in turn can affect their caregiving behaviour toward their baby, and can lead to healthier families 18 19 . On the other hand, a negative or traumatic birth experience has been found to be associated with serious multifaceted and far-reaching consequences for mothers/birthing parents, partners, and the whole family, including an increased risk for mental health problems such as postpartum depression, postpartum anxiety, and childbirth-related posttraumatic stress disorder (CB‑PTSD), impaired parent-child bonding and breastfeeding behaviour, poorer couple relationship satisfaction, lower sexual functioning and satisfaction, and secondary fear of childbirth affecting subsequent pregnancy intentions 12 17 20 21 22 23 24 25 . Also, child development may be impeded through indirect pathways, for example, an increased risk for infant regulatory problems (such as sleeping and feeding problems) have been found for infants of mothers with CB-PTSD symptoms 26 27 . The potential consequences at both the individual and family level but also the societal level, including the social and economic costs associated with mental health disorders and healthcare utilisation, highlight the relevance of the subjective birth experience as a significant public health issue. Avoiding or delaying subsequent pregnancies due to negative childbirth experiences may even affect fertility rates 28 , which is particularly relevant for countries where birth rates are declining, as it is the case in Germany where the current study takes place.

Existing findings on some outcomes (e.g., parental mental health, parent-child bonding, couple relationship satisfaction) show inconsistencies and need further investigation. There is some evidence that the subjective birth experience may not remain stable over time for all parents (e.g., 29 30 ). However, there are also hints that a negative subjective birth experience will never become a positive one 31 32 . While previous research mostly focused on short-term implications of mothers’ birth experiences, there are only very few studies 12 17 that have analysed the long-term consequences beyond the first few months postpartum and possible changes of the subjective birth experience over time.

### Predictors of the subjective birth experience

Given the far-reaching consequences associated with the subjective birth experience, identifying factors that influence the subjective birth experience is crucial, particularly those that are easily modifiable in order to promote a positive birth experience and mitigate the risk of negative consequences. Overall, the subjective birth experience is influenced by an interplay of physical, psychological, environmental, social, organisational, and policy factors 33 . A wide range of individual, birth-related, and care-related factors has been investigated as predictors of a positive or negative subjective birth experience of mothers/birthing parents. According to a systematic review of predictors of mothers’ subjective birth experience 1 , birth-related factors that have been associated with a more negative subjective birth experience include birth complications and excessive or unplanned medical interventions (e.g., emergency caesarean sections, instrumental vaginal birth using forceps), prolonged labour, severe pain, and giving birth in a hospital (compared to an out-of-hospital or home birth). Conversely, a spontaneous vaginal birth and perceived control during childbirth have been associated with a positive subjective birth experience in multiple studies 1 . Meeting birth expectations may play a particularly important role, as a discrepancy between birth expectations and the actual birth experience (e.g., preferring a spontaneous vaginal birth but ultimately having a caesarean section, or vice versa 34 ) can have a negative impact on mothers’/birthing parents’ satisfaction with the birth 35 36 . The presence of and support from the partner during childbirth has consistently been found to predict a more positive subjective birth experience, while the partner’s absence or lack of support during childbirth was associated with a more negative subjective birth experience 1 . With regard to individual factors, inconsistent results have been reported for demographic variables such as maternal age and educational level in relation to the subjective birth experience 1 . Parous women tend to have a more positive subjective birth experience in some studies 37 38 39 , while others did not find a significant association for parity 40 41 . Regarding psychological factors, fear of childbirth as well as symptoms of depression or anxiety prior to or during pregnancy have been repeatedly linked to a more negative subjective birth experience 1 .

Similar to mothers/birthing parents, the scarce research on predictors of partners’ birth experiences revealed the most consistent results for the mode of birth and whether the first child was expected. Compared to a spontaneous vaginal birth, an instrumental vaginal birth and an unplanned or emergency caesarean section were associated with a more negative birth experience 4 16 29 37 42 43 . Also, the risk of experiencing the birth as negative is higher among first-time fathers, possibly because their expectations are more likely to be unmet 10 . Accordingly, expectations also seem to be particularly important for partners’ birth experiences.

### The role of obstetric health care staff during childbirth

Besides (objective) birth-related and individual factors, the interactions between the (expectant) mother/birthing parent and the obstetric health care staff overseeing the childbirth play a key role in the subjective birth experience, which is highlighted in numerous studies and position papers (e.g., 44 45 ). Perceiving the midwives and obstetricians present at childbirth as (emotionally) supportive and professional, having a good relationship with them, and receiving adequate information is related to a more positive subjective birth experience in mothers 1 . On the other hand, not feeling heard or seen during childbirth or being treated with disrespect is associated with a more negative subjective birth experience 1 . With regard to partners, the extent to which they are involved in the birth process and the way in which communication with and support from midwives is organised seem to be crucial for their subjective birth experience 46 47 48 . However, partners are often not sufficiently included in their role by the obstetric health care staff and may feel like outsiders in the delivery room 49 50 51 , which is related to a more negative birth experience 52 53 . Several studies emphasise that the obstetric health care staff present at childbirth can even buffer the negative effects of a (medically) difficult birth for both mothers/birthing parents (e.g., 29 54 55 ) and partners 46 47 56 , making the overall experience positive even in the face of challenges or complications 57 .

### Mistreatment and disrespect during childbirth

During the last decade, attention toward the issue of mistreatment during childbirth, also referred to as “obstetric violence”, has increased worldwide. Already in 2014, the World Health Organization stated that “every woman has the right to the highest attainable standard of health, which includes the right to dignified, respectful health care” 58 and called for the prevention and elimination of disrespect and abuse during facility-based childbirth as a global public health issue. According to Bohren, Vogel, Hunter, et al. 59 , there are various types of mistreatment during childbirth. These include explicit forms such as physical, sexual, or verbal abuse, but also subtle forms that may be overlooked or not directly considered as violence such as stigma and discrimination, the failure to meet professional standards of care, or poor rapport between women and providers, as well as certain health care system conditions and constraints 59 . Existing evidence suggests that mistreatment during childbirth is more pervasive in low-income and middle-income countries 60 . Knowledge on the situation in high-income countries is limited, but there are first studies indicating that it also occurs in high-income countries despite their advanced health care systems 61 62 63 64 . A recent U.S. study including a representative sample of 4458 mothers/birthing parents who gave birth in 2020 reported an overall mistreatment rate of 13.4% 65 . No systematic data on the frequency and types of mistreatment are available in Germany, but initial research indicates that the issue may be relevant there as well 66 . In a non-representative online survey with 2045 mothers 67 , more than three quarters reported having experienced one or several forms of mistreatment, with performing obstetric procedures without consent, physical abuse (e.g. painful vaginal examinations), and violation of physical privacy (i.e., having other people in the room without consent) being the most prevalent. However, given that the participants were recruited through platforms that are more likely to be used by mothers/birthing parents who have experienced mistreatment during childbirth, the authors point towards a selection bias in their study that aimed to validate a survey tool measuring disrespect and abuse during childbirth in Germany. In another recent study concerning violated birth integrity during the COVID-19 pandemic, 14% of the participants felt physically, verbally, or emotionally abused 68 .

### Person-centred care in obstetric health care during childbirth

One promising approach to encounter mistreatment during childbirth that is included in the recommendations for a positive childbirth experience by the World Health Organization 45 and has been added to the new S3 guideline “Vaginal Birth at Term” in 2020 69 , is person-centred care (PCC). PCC comprises a strong relationship between care provider and patient, shared-decision making, and effective communication 70 . In the perinatal context, PCC can been defined as “providing reproductive health care that is respectful and responsive to individual women and their families’ preferences, needs, and values, and ensuring that their values guide all clinical decisions” 71 , based on a general definition of PCC from the Institute of Medicine 72 .

Higher levels of PCC in obstetrics have been shown to improve childbirth outcomes by lowering the risk of complications for both the mother/birthing parent (e.g., vaginal bleeding or mastitis) and the newborn (e.g., jaundice or breathing difficulties 73 74 ). Further, a decreased risk for symptoms of postpartum depression or CB-PTSD has been reported 74 75 . However, evidence specifically focusing on the concept of PCC during childbirth is limited to a few international studies. Research that explicitly investigates the relationship between PCC during childbirth and the subjective birth experience, as well as potentially influencing factors is limited. In one Iranian study, respectful maternity care was significantly correlated with a more positive childbirth experience in mothers who gave birth vaginally 76 . Other studies focused on the satisfaction with care received during childbirth rather than the overall subjective birth experience 77 . As PCC includes domains such as autonomy, social support, and supportive care 78 that have been linked to a more positive birth experience, it is likely that the provision of higher levels of PCC during childbirth contributes to a more positive subjective birth experience. Although PCC is considered to be a broad concept, encompassing more than just the absence of mistreatment during childbirth 79 , PCC and mistreatment are closely linked 80 . Therefore, the implementation of PCC in obstetrics may not only be a driver of positive birth experiences but may also prevent mistreatment.

As the staff bear the responsibility for providing respectful and person-centred care in obstetrics 81 , it is essential that they are involved in research in order to improve the implementation of person-centred approaches into the care during childbirth and, in turn, to improve parents’ subjective birth experience. However, a high percentage of obstetric health care staff is not sufficiently familiar with the concept of PCC and its health-related benefits (e.g., 82 ). Furthermore, studies from low- and middle-income countries show that the perceptions of obstetric health care staff and expectant mothers/birthing parents regarding ethically desirable obstetric care can be highly discordant 83 84 . Consequently, there is a need for research that takes a closer look at the perceptions and attitudes of obstetric health care staff, as well as the contributing factors and barriers in their daily work that facilitate or hinder the provision of PCC. Additionally, it remains unclear what role obstetric health care staff attribute to themselves with regard to the subjective birth experience of parents.

### Research gaps and aims of the current project

In sum, several research gaps need to be addressed in order to achieve a comprehensive understanding of the subjective birth experience. Although there is some evidence from international studies on potentially influencing factors of the mothers’/birthing parents’ subjective birth experience, there is a lack of studies that consider multiple predictors simultaneously and their possible interplay. Also, even if studies include both parents, data analyses are usually conducted at the individual level, although dyadic analyses that take into account interdependencies within the couple may be particularly beneficial for certain research questions 85 . Data on mistreatment during childbirth and its impact on the subjective birth experience and other outcomes is limited, as is research on PCC as a promising approach to foster positive birth experiences, and there is a lack of research that involves obstetric health care staff as a target group and their personal perspectives in addition to those of parents.

The present interdisciplinary research project **RESPECT** (“A Prospective Mixed-Methods- **RE** search Project on **S** ubjective Birth Experience and **PE** rson-centred **C** are in Paren **T** s and Obstetric Health Care Staff”), consisting of a prospective cohort study (main study RESPECT _PARENTS_ ) together with its qualitative sub-study RESPECT _PARENTS-TALK_ and the additional study branch RESPECT _STAFF_ , aims to systematically investigate the subjective birth experience of (expectant) parents and its related factors before, during, and after birth. By including the perspectives of multiple target groups, i.e., (expectant) mothers/birthing parents, partners, as well as the obstetric health care staff, and by integrating quantitative and qualitative approaches, RESPECT will contribute to an enhanced and more comprehensive understanding of the subjective birth experience, its predictors, short- and long-term consequences, as well as its development over time, thus extending the literature in many respects.

**Fig. 1:**
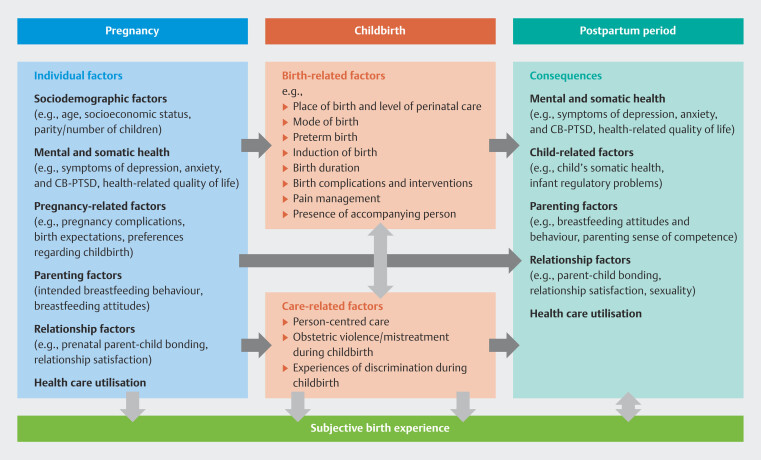
Simplified theoretical framework upon which the main study RESPECT _PARENTS_ is based.

Fig. 1 shows a variety of individual, birth-related, and care-related factors during pregnancy and childbirth that potentially affect the parents’ subjective birth experience as well as possible consequences in the postpartum period. Based on this theoretical framework, the key research questions of the quantitative main study RESPECT _PARENTS_ are as follows:

1. What individual, birth-related, and care-related factors predict a positive or negative subjective birth experience for (expectant) parents at eight weeks, six months, and 24 months postpartum?
2. What expectations do expectant parents have about childbirth and what factors are associated with these expectations?
3. Does a discrepancy between birth wishes and plans and the objective birth experience predict the subjective birth experience of (expectant) parents?
4. Do mothers/birthing parents and partners differ in their subjective birth experiences, and if so, what factors determine these differences?
5. Are parents exposed to mistreatment during childbirth, and if so, to what extent and what kind of mistreatment?
6. Does parents’ subjective birth experience evolve from eight weeks to six months and 24 months postpartum, and if so, how?
7. Does a negative subjective birth experience predict mental health problems and/or poor health-related quality of life of parents at eight weeks, six months, and/or 24 months postpartum?
8. What are the associations between parents’ subjective birth experience, obstetric violence/mistreatment during childbirth, breastfeeding beliefs and behaviour, and parent-child bonding at eight weeks and six months postpartum?
9. What are the associations between parents’ subjective birth experience, relationship satisfaction, sexuality, and future family planning at eight weeks, six months, and 24 months postpartum?

In addition, the following research questions will be investigated exploratively as part of the qualitative sub-study RESPECT _PARENTS-TALK_ :

1. What role do parents attribute to obstetric health care staff in relation to their birth experience?
2. What factors related to the care provided by obstetric health care staff do parents perceive as beneficial or detrimental to their birth experience?
3. What are parents’ expectations, needs, and wishes concerning the care by obstetric health care staff?

The research questions of the additional study branch RESPECT _STAFF_ include:

1. What are obstetric health care staff’s perceptions about a positive and negative subjective birth experience, particularly with regard to their own impact on those experiences?
2. What knowledge, beliefs, and attitudes do obstetric health care staff have regarding the concept of PCC?
3. What institutional and psychological aspects of clinical routines act as facilitators or barriers for obstetric health care staff’s own ability to offer PCC?

## Methods

### Overall design and procedure

**Fig. 2:**
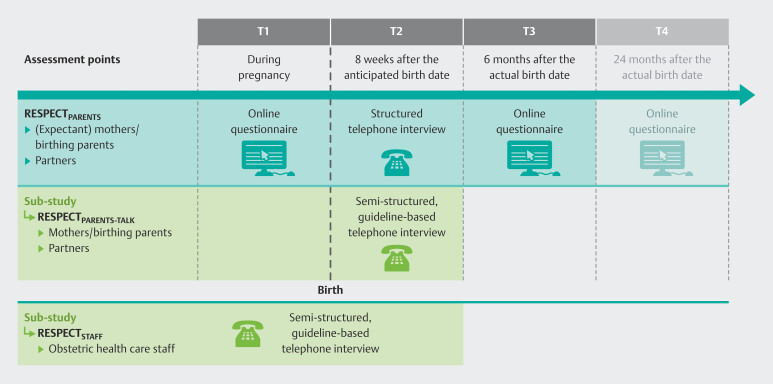
Target groups, assessment points, and methods of the quantitative main study RESPECT _PARENTS_ as well as its qualitative sub-study RESPECT _PARENTS-TALK_ , and the additional study branch RESPECT _STAFF_ .

The interdisciplinary research project **RESPECT** (A Prospective Mixed-Methods- **RE** search Project on **S** ubjective Birth Experience and **PE** rson-centred **C** are in Paren **T** s and Obstetric Health Care Staff) consists of two study branches, targeting (expectant) parents on the one hand (RESPECT _PARENTS_ and RESPECT _PARENTS-TALK_ ) and obstetric health care staff on the other hand (RESPECT _STAFF_ ). The overall design of the project is presented in Fig. 2 . The quantitative and qualitative components of the study will be described in detail below.

RESPECT is a joint project of the MSH Medical School Hamburg and the Technische Universität Dresden, Germany, specifically the Department of Gynecology and Obstetrics at the University Hospital Dresden in cooperation with the Institute and Policlinic of Occupational and Social Medicine of the Technische Universität Dresden. It is supported by an Advisory Board consisting of national and international experts in the field of perinatal health research and/or care who have been involved from the planning stage and continuously support the study team at key milestones throughout the project. To ensure that parents’ perspective is also reflected, the parents’ association Mother Hood e. V., dedicated to safe childbirth and improved maternity care, is represented in the RESPECT Advisory Board.

The RESPECT project is pre-registered in the Open Science Framework (OSF; https://doi.org/10.17605/OSF.IO/CAQG7 ).

### RESPECT _PARENTS_

The prospective cohort study RESPECT _PARENTS_ (main study) targets a community sample of (expectant) mothers/birthing parents as well as partners expecting a child in the area of Dresden, Germany. Inclusion criteria are

- a current pregnancy (own or partner),
- being of legal age (≥ 18 years),
- being a resident in or planning childbirth in the Dresden area,
- having sufficient German or English skills to complete the study questionnaires, and
- giving informed consent to participate in the study.

Expectant parents can participate either as a couple or alone, regardless of whether they are currently in a permanent relationship or not.

RESPECT _PARENTS_ currently comprises three assessment points starting with T1 during late pregnancy (at least 24 ^th^ gestational week), T2 at eight weeks after the anticipated birth date (end of puerperium), and T3 at six months after the actual birth date. Further, an additional assessment point T4 is planned at 24 months after the actual birth date, depending on subsequent funding (see Fig. 1 ). The time points of T3 and T4 were chosen in order to monitor possible medium- and long-term changes in parents’ subjective birth experience and its health-related consequences over time.

Three surveys (T1, T3, presumably T4) are conducted via online questionnaires and one survey (T2) is conducted as a structured telephone interview. The latter was chosen over an online questionnaire because the personal contact at this time point, relatively soon after birth, is expected to increase response rates, result in higher data quality, minimise missing values (e.g., with regard to incidences and birth complications that are partly not easily understood by the participants), and strengthen participants’ commitment to participate in follow-up assessments 86 . Missing one assessment point does not result in study exclusion or dropout (e.g., fail to participate in T2, but complete T3). Missing relevant objective information (e.g., date of birth, mode of birth, or basic sociodemographic variables) will be collected at the subsequent assessment point. Parents whose children have been stillborn are excluded from T2 onwards. Although it is important to not generally exclude parents with stillborn children 87 , the content and design of the current study do not allow the special situation of parents experiencing stillbirth to be adequately addressed.

Recruitment for RESPECT _PARENTS_ ran from April 2023 to December 2024. Expectant parents were recruited primarily through three different recruitment channels. Firstly, two study nurses addressed expectant parents during their prenatal appointments (e.g., for pre-registration for childbirth or for prenatal care or diagnostics) or at the inpatient prenatal treatment ward at the University Hospital Dresden. Secondly, student assistants approached expectant parents at birth information events at all other maternity hospitals and a freestanding birth centre in Dresden, including different levels of perinatal care 3 88 as well as midwife-led settings and hospitals certified as baby-friendly according to the “Baby-Friendly Hospital Initiative” by the WHO and UNICEF 89 90 . Thirdly, additional recruitment strategies included recruitment in settings frequented by expectant parents (e.g., gynaecologists’ and midwives’ practices, pregnancy counselling services, or family centres), as well as through social media channels (Facebook, Instagram).

Interested expectant parents accessed the study website via a link or Quick Response (QR) code placed on the recruitment materials and subsequently received written information about the study. After giving informed consent for participation, they completed a short screening questionnaire and reported contact information. If inclusion criteria are fulfilled, participants receive access to the first online questionnaire (T1) either immediately (if they have already reached the 24 ^th^ gestational week or beyond) or at a later stage when the pregnancy has progressed to 24 weeks. Participants receive no payment for their study participation but a small incentive at study registration (in the case of in-person recruitment through the study nurses or student assistants), at T3, and presumably at T4. In addition, vouchers and small gifts are raffled quarterly as part of a lottery.

As thinking and talking about pregnancy and childbirth during the interview or online assessment may be emotionally upsetting for some parents, especially for those who have had a negative birth experience, or they may become aware of potential problems and the need to seek help, a list of services in the Dresden area that can support them is offered to participants at the end of each assessment.

All persons performing the structured telephone interviews at T2 undergo an extensive interview training, follow interview guidelines, and receive regular supervision, aiming to ensure a coherent procedure and coding of answers across all interviewers. When conducting the interviews, all interviewers read exactly the same instructions and questions of the quantitative questionnaires to the participants and record their answers directly on the computer.

### RESPECT _PARENTS-TALK_

In the qualitative sub-study RESPECT _PARENTS-TALK_ , a sub-sample of participants of the RESPECT _PARENTS_ main study is interviewed in semi-structured, guideline-based interviews about their subjective birth experience, particularly with regard to the role of obstetric health care staff. Mothers/birthing parents and partners are eligible for RESPECT _PARENTS-TALK_ if they

- participate in the main study RESPECT _PARENTS_ ,
- complete the structured telephone interview at eight weeks after the anticipated birth date (T2), and
- consent to participate in RESPECT _PARENTS-TALK_ .

Parents with twins or multiples are excluded from participation in this sub-study as multiple births present higher risk births that require special care that can deviate significantly from the norm and typically require more interventions (e.g., 91 ).

After completing the structured telephone interview at T2, mothers/birthing parents and partners from the main study are selected based on their responses in the interview. They are then contacted again and invited to participate in RESPECT _PARENTS-TALK_ . As soon as informed consent for this sub-study is obtained, the interviews are conducted via videoconference, using the software “Big Blue Button”. Videoconferencing has been described as an adequate alternative to face-to-face interviews when organisational challenges are present 92 , as it might be the case for parents of newborns for whom attending the interview from home is easier and more convenient. Compared to telephone interviews, videoconferencing is preferred for longer interviews and more personal topics because it creates a pleasant atmosphere and rapport.

Eligible mothers/birthing parents and partners are screened and selected for participation based on pre-defined screening criteria. These criteria were developed by the study team, informed by the theoretical background of RESPECT, in collaboration with the RESPECT Advisory Board. Ultimately, items reflecting the developed criteria were selected from the quantitative surveys of the main study RESPECT _PARENTS_ . The developed screening criteria can be classified at three distinct levels of relevance for the selection process: main criteria, secondary criteria, and additional criteria (see Supplementary **Table S1** ). The main criteria (i.e., perception of interactions with obstetric health care staff, subjective birth experience, mode of birth, and number of interventions) are the most important criteria and must be covered in different manifestations in the sub-sample in order to obtain a diverse sample. The secondary criteria (e.g., number of previous births, birth expectations, place of birth, or birth complications) are at the second level of importance for the screening process, meaning that as many of them as possible should be covered in different manifestations in the sub-sample. The third level of screening criteria is called “additional criteria” (e.g., country of birth/mother tongue, age, educational level, or pregnancy complications). Covering them in different manifestations in the sub-sample is optional, but potentially valuable if the data allow for it. By applying purposive sampling, the aim is to select a sample that is as diverse as possible with respect to the developed screening criteria.

### RESPECT _STAFF_

Within the additional study branch RESPECT _STAFF_ , obstetric health care staff are interviewed once nationwide about their own role in parents’ birth experience and their knowledge and attitudes regarding PCC in obstetrics. The inclusion criteria for RESPECT _STAFF_ are

- being a midwife or obstetrician (including midwifery students and residents),
- currently working in childbirth care,
- being of legal age (≥ 18 years),
- having sufficient language skills in German,
- living and working in Germany, and
- giving informed consent to participate in the sub-study RESPECT _STAFF_ .

Obstetric health care staff are recruited independently of the main study through invitation e-mails that are sent to the secretaries’ offices of the obstetric departments of selected maternity hospitals in Germany, to freestanding birth centres, and to some home birth midwives. A pre-selection of maternity hospitals was made to cover a broad spectrum of regions (ideally each federal state) and different levels of perinatal care. The invitation e-mails include the request to forward the invitation to the entire staff or to interested colleagues. Additionally, some obstetric health care staff are recruited directly by personal invitation e-mails. This includes obstetric health care staff who have in the past spoken publicly about topics relevant to PCC in obstetrics, as well as obstetric health care staff who have been suggested as participants by members of the RESPECT Advisory Board, national expert societies, or by other participants of RESPECT _STAFF_ . Efforts are made to create a diverse sample in terms of age, professional experience, type of institution the midwives and obstetricians work in, and their attitudes and beliefs about childbirth and obstetric care (i.e., purposive sampling). To achieve this, interested obstetric health care staff are asked to self-register on a website that includes questions regarding the inclusion criteria, contact information, a short questionnaire consisting of selected items from the “Nurse Attitudes and Beliefs Questionnaire – Revised” (NABQ-R) 93 , and a tool to select and book an interview appointment. The items from the NABQ-R were chosen in close cooperation with the RESPECT Advisory Board. Recruitment will continue until a diverse sample is achieved with respect to these factors. As is the case for RESPECT _PARENTS-TALK_ , the interviews will be conducted via videoconference using the software “Big Blue Button” in order to create a pleasant atmosphere for the discussion, and to make it possible for obstetric health care staff from all over Germany to take part.

### Materials

RESPECT is characterised by a mixed-method approach combining quantitative questionnaires (main study RESPECT _PARENTS_ ) and qualitative semi-structured, guideline-based interviews (RESPECT _PARENTS-TALK_ and RESPECT _STAFF_ ).

Study data are collected and managed using Research Electronic Data Capture (REDCap) 94 95 , a secure web-based application designed to facilitate data collection in research studies, hosted at the “Koordinierungszentrum für Klinische Studien”, an institution of the Faculty of Medicine of the Technische Universität Dresden, Germany. Within the main study, automated invitation e-mails and reminders are sent to participants via REDCap when an online assessment (T1, T3, presumably T4) is due. In order to schedule the structured interviews at eight weeks postpartum (T2), participants are contacted via phone and/or e-mail.

In addition, within RESPECT _PARENTS-TALK_ and RESPECT _STAFF_ , the interviews are audio-recorded and subsequently transcribed verbatim in German.

#### Questionnaires

Within the main study RESPECT _PARENTS_ , a number of quantitative questionnaires are included to measure the subjective birth experience as well as (potentially) related factors before, during, and after childbirth. An overview of all constructs assessed and the instruments used at the respective assessment points is provided in Table 1 . Topics covered are sociodemographic factors, somatic factors (e.g., health-related quality of life), mental health (e.g., symptoms of depression, anxiety, and CB-PTSD, prior exposure to traumatic life events), pregnancy and birth-related factors (including birth expectations and experiences, PCC, as well as obstetric violence/mistreatment and discrimination during childbirth), child-related factors (e.g., child’s somatic health, parent-child bonding, and breastfeeding), relationship factors (e.g., relationship satisfaction, sexuality, and social support), and health care utilisation.

**Table:** **Table 1** Constructs and instruments in the main study RESPECT _PARENTS_ .

Constructs	Instruments	T1	T2	T3	T4
**Sociodemographic factors**					
Sociodemographics (e.g., age, nationality, mother tongue, marital status, size of household)	Adapted questions derived from the German National Cohort 96 , the DREAM study 9 , and self-generated questions	x			
Socioeconomic status (SES)	SES-Index based on the following variables from the study KiGGS Welle 2 97 : educational qualification, occupational status, and income situation 98	x			x
Biological sex and gender identity	Adapted questions derived from the “Assessment of anti-discrimination data in representative repeated surveys” (“Erhebung von Antidiskriminierungsdaten in repräsentativen Wiederholungsbefragungen”) 99	x			
Membership of a discriminated population group	Adapted questions derived from the European Social Survey (ESS; Welle 7; German version) 100	x			
**Somatic factors**					
Height and weight	Adapted questions derived from the DREAM study 9 based on the Akershus Birth Cohort 101 102	x		x	x
Health-related quality of life	Short-Form Health Survey (SF-8) 103 104	x		x	x
**Mental health factors**					
Symptoms of depression	Edinburgh Postnatal Depression Scale (EPDS) 105 106	x	x	x	x
Symptoms of anxiety	Generalised Anxiety Disorder 7 (GAD-7) 107 108	x	x	x	x
Former and current mental disorders (depression, anxiety, posttraumatic stress disorder) and treatments	Adapted questions derived from the DREAM study 9		x	x	x
(Prior) exposure to traumatic life events	Selected life events of the Life Stressor-Checklist Revised (LSC-R) 109 with focus on history of violence and abuse	x			
Stressful experiences in the health care system	NorVold Abuse Questionnaire (NorAQ) 110	x			
Symptoms of childbirth-related posttraumatic stress disorder ^a^	City Birth Trauma Scale (City BiTS) 111 112	x	x		x
**Pregnancy factors**					
Birth expectations (including fear of childbirth)	Wijma Delivery Expectancy/Experience Questionnaire (W-DEQ), version A 113 114 115	x			
Preferences regarding childbirth (e.g., place of birth, mode of birth, pain management, accompanying persons)	Self-generated questions	x			
(Objective) Information on pregnancy (esp. pregnancy complications)	Maternity records (“Mutterpass”) 116 , adapted questions derived from the DREAM study 9 , the INVITE study 86 , and self-generated questions		x		
**Birth-related factors**					
(Objective) Information on childbirth (esp. birth complications, mode of birth, birth duration, pain management, skin contact after birth)	Maternity records (“Mutterpass”) 116 , adapted questions derived from the Birth Integrity Study (“Geburt und Integrität”; Universität Bielefeld, 2021), the DREAM study 9 , the INVITE study 86 , and self-generated questions		x		
Subjective birth experience	Wijma Delivery Expectancy/Experience Questionnaire (W-DEQ), version B 113 117		x	x	x
**Care-related factors**					
Person-centred care during childbirth	Women’s Perspection-Respectful Maternity Care (WP-RMC) questionnaire 118		x		x
Obstetric violence/mistreatment during childbirth	Adapted mistreatment items according to Limmer et al. (2023) 67		x		
Experiences of discrimination during childbirth	Adapted discrimination list according to Limmer et al. (2023) 67		x		
**Child-related factors**					
(Objective) Information on child’s health (e.g., birth weight and height, Apgar scores, medical complications after birth, treatment in hospitals, chronic illnesses)	Medical records (“Kinderuntersuchungsheft”) 119 and adapted questions derived from the DREAM study 9		x		
Subjective general health status	Self-generated question		x	x	x
Infant regulatory problems (excessive crying, feeding and sleeping problems)	Questionnaire on crying, feeding and sleeping disorders (German: Fragebogen zum Schreien, Füttern, Schlafen; SFS) 120			x	
Early childhood behavioural and emotional problems	Early Childhood Screening Assessment (ECSA; German version: Screening Frühe Kindheit; SFK) 121 122				x
**Parenting factors**					
Breastfeeding attitudes	Beliefs about Breastfeeding Questionnaire (BAB-Q) 123 and one additional self-generated question	x	x	x	x
Intended and actual breastfeeding behaviour	Questions derived from the Infant Feeding Practices Study II 124	x	x	x	x
Parenting Sense of Competence	Parenting Sense of Competence Scale Revised (PSOC-R) 125 126				x
Parental Communication Expectations	Adapted version of the Revised Family Communication Pattern (RFCP) instrument 127				x
**Relationship factors**					
Prenatal parent-child bonding	13-items version of the Maternal Antenatal Attachment Scale (MAAS) 128 129 130 /Paternal Antenatal Attachment Scale (PAAS) 128 129 131	x			
Postpartum parent-child bonding	Postpartum Bonding Questionnaire (PBQ) 132 133		x	x	x
Relationship satisfaction	Relationship Satisfaction Scale, short version (RS5) 134	x	x	x	x
Sexual activity and satisfaction	Self-generated questions			x	x
Impact of childbirth on sexuality	Sexual Function Questionnaire’s Medical Impact Scale (SFQ-MIS) 135			x	x
Family planning (i.e., wishes and plans regarding number of biological children)	Self-generated questions based on the FReDa Welle 1 136	x		x	x
Social support	The Oslo Social Support Scale (OSSS-3) 137	x	x	x	x
**Health care utilisation**					
Utilisation of psychological treatment or psychotherapy	Adapted questions derived from the INVITE study 86		x	x	x
Pre- and postpartum utilisation of support services (e.g., early help and pre- and postpartum care by midwives)	Adapted questions derived from the INVITE study 86		x	x	x
Notes: DREAM = Dresden Study on Parenting, Work, and Mental Health (“ **DR** esdner Studie zu **E** lternschaft, **A** rbeit und **M** entaler Gesundheit”); INVITE = **IN** timate partner **VI** olence care and **T** reatment pr **E** ferences in post-partum women; T1 = late pregnancy (at least 24 ^th^ gestational week); T2 = eight weeks after anticipated birth date; T3 = six months after actual birth date; T4 = 24 months after actual birth date ^a^ At T1 related to potential previous births, at T2 related to the index birth.					

Standardised and validated instruments with good psychometric characteristics, preferably available in both English and German, were chosen whenever possible. If the German version of a selected questionnaire was not available, the English items and instructions were translated into German by two members of the study team and a consent version was developed, which was back-translated by a native speaker. Short instruments were favoured to minimise the burden on participants in terms of time to complete the surveys. Because of the target group of the main study, instruments generally needed to be applicable to both (expectant) mothers/birthing parents and partners. Ideally, a validated version for both groups was available (e.g., City Birth Trauma Scale [City BiTS] 111 112 138 ). Furthermore, instruments were appropriate if they took into account the specific circumstances of the perinatal period (e.g., using the Edinburgh Postnatal Depression Scale [EPDS] 105 106 ). Finally, a selection of possible instruments was discussed with the RESPECT Advisory Board, especially instruments assessing PCC and obstetric violence/mistreatment during childbirth as well as parent-child bonding.

Subjective birth experience, the core construct of the RESPECT study, as well as birth expectations are measured using the Wijma Delivery Expectancy/Experience Questionnaire (W-DEQ) 113 . Version A is administered during pregnancy and asks participants how they imagine their childbirth will be like and how they think they will feel, while version B is administered after childbirth and asks about the actual thoughts and feelings participants had during childbirth. Both versions comprise 33 statements, each to be answered on a six-point Likert scale (e.g., “0 = not at all fantastic” to “5 = extremely fantastic”). The maximum possible total score is 165, with higher scores indicating more negative expectations (version A) or a more negative subjective birth experience (version B). A sum score ≥ 85 indicates intense, clinically relevant fear of childbirth (version A) or a very negative birth experience (version B), respectively. By using both version A and version B within the study, it is possible to compare the birth expectations and actual birth experiences. The W‑DEQ is a widely used assessment tool in the field of perinatal psychology and obstetrics. It has been extensively validated and used in research and clinical settings worldwide, with excellent psychometric properties reported 113 139 140 . The instrument was originally developed for use with (expectant) mothers, but has also been used with (expectant) fathers 141 142 .

The second core construct of RESPECT, PCC, is assessed using the Women’s Perception-Respectful Maternity Care (WP-RMC) questionnaire 118 . The instrument was developed in Iran and validated among women with low-risk pregnancies who gave birth vaginally to healthy, normal-weight babies in Teheran. The WP‑RMC consists of 19 items loading on three factors: “Providing Comfort”, “Participatory Care”, and “Mistreatment”. Unlike other proposed instruments, the WP-RMC covers most of the dimensions of PCC (dignity, autonomy, privacy/confidentiality, communication, social support, supportive care, trust, and health facility environment) according to the “Person-Centred Care Framework for Reproductive Health Equity” 78 . Its items ask for the mother’s/birthing parent’s subjective assessment of the treatment by the care providers during birth (e.g. “My care provider treated me in a friendly manner”). Furthermore, the WP-RMC includes an item on the inclusion of birth partners (“I was allowed to have the companion of my choice during labour”), which is essential to PCC. Although there are several other scales available that assess related concepts such as respectful maternity care, mistreatment, discrimination, satisfaction with childbirth care, autonomy in decision-making, or perceived control (e.g., 143 144 145 ), no validated instrument for measuring PCC in high-income countries like Germany exists. Although the WP-RMC is neither available in German nor validated in a German sample, we considered the WP-RMC to be the most applicable to the German health care system and most suitable for the projects’ objectives compared to other measures.

Although one of the three subscales of the WP-RMC is mistreatment, events of obstetric violence/mistreatment during childbirth are measured in greater detail using an additional short questionnaire including 13 single items. The items were derived from a validated instrument on disrespect and abuse during childbirth in Germany 67 and reflect different forms of obstetric violence/mistreatment according to the typology by Bohren, Vogel, Hunter, et al. 59 , e.g., physical abuse, verbal abuse, or the failure to meet professional standards of care. In contrast to the original version by Limmer, Stoll, Vedam, et al. 67 , physical and sexual violence are recorded using two separate items within the present study, resulting in one more item for the total scale. Each item is answered using a dichotomous yes-no format, depending on whether the situation described was experienced during childbirth or not (e.g., “interventions [e.g., episiotomy, caesarean section, oxytocin infusion, amniotomy, drug injection, venous access] were conducted without your consent”). A mistreatment-index (MIST-I) can be calculated from the sum of the items that were answered with “yes” 67 . Also, in case of interventions without consent, an additional follow-up question asks about what kind of interventions this pertained to.

Finally, discrimination during childbirth is measured using an adapted list of items from the validated instrument by Limmer, Stoll, Vedam, et al. 67 . In addition to the nine items of the original version with different possible reasons to be discriminated (i.e., (1) race, ethnicity, cultural background or language, (2) sexual orientation or gender identity, (3) handicap/chronic disease, (4) HIV status, (5) age, (6) overweight, (7) socioeconomic situation, (8) type of health insurance or lack of insurance, and (9) difference in opinion with caregivers about the right care), the following reasons were added: religious affiliation, mental disorder, alcohol and/or drug use, COVID-19 infection, underweight, number of older children, being a single mother, as well as an “other reasons” category. Consequently, a total of 17 reasons were listed, each of which are confirmed or denied according to the participants’ actual experiences during birth, using a dichotomous yes-no format.

#### Semi-structured, guideline-based interviews

As part of the qualitative sub-study RESPECT _PARENTS-TALK_ , a sub-sample of parents is surveyed to explore their perceptions of the role and impact of obstetric health care staff during childbirth. At the beginning of the semi-structured, guideline-based interviews, participants are generally asked to talk about the birth of their child and their subjective birth experience. Following this initial description, the interview guide includes questions on the following topics: the dimensions of PCC in obstetrics (e.g., respect, consent, communication, family involvement, personal preferences, and recognition of physical and psychological needs), and the expectations, needs, and wishes concerning the care by obstetric health care staff.

Within RESPECT _STAFF_ , the interview with obstetric health care staff starts with several questions about their daily work, views on the subjective birth experience, experiences with the expectations of and the relationship with the families they care for in obstetrics, and their personal experiences with PCC. The experts are then presented with three written case vignettes, which are subjective reports written by mothers on how they have experienced childbirth, and asked for their subjective assessment of the situations described. Using case vignettes is helpful to create distance between the discussed issues and the participant when topics are sensitive, as it is the case in the context of mistreatment and PCC during childbirth 146 . Several written reports were gathered from Facebook groups, blogs, and other online platforms where mothers share their experiences during childbirth. The reports contained descriptions of different positive and negative experiences with obstetric health care staff, agreed upon with representatives of the organisation Mother Hood e. V. Out of 10 preselected reports, the three most suitable ones (i.e., one positive and two negative reports) were selected in cooperation with the RESPECT Advisory Board, taking into account feedback obtained after two pilot interviews. Finally, the experts are asked to talk about aspects of their daily work that, from their own perspective, facilitate or hinder the provision of PCC in practice.

### Planned data analysis

#### Quantitative data analysis

In the quantitative analyses, sum scores and/or mean scores will be calculated to reflect the subjective birth experience, as well as the mothers’/birthing parents’ experiences of support and person-centredness offered by the obstetric health care staff. Univariate frequencies will be used to determine the prevalence of obstetric violence/mistreatment and experiences of discrimination during childbirth. Correlation analyses will be used to examine associations between different variables, such as the birth experiences of mothers/birthing parents and partners. Independent sample *t* -tests or χ²-tests will be used to examine differences between mothers/birthing parents and partners (e.g., regarding the subjective birth experience, symptoms of CB-PTSD, and parent-child bonding).

The main goal of the quantitative main study, however, is to identify factors that predict the subjective birth experience and to examine the potential short- and long-term effects of positive or negative birth experiences on mothers/birthing parents and their partners. These key research questions will be addressed using simple and multiple linear regression analyses (e.g., investigating the influence of the subjective birth experience on parental symptoms of depression or parent-child bonding), moderator and mediator analyses, and structural equation modeling. In addition, the study provides the opportunity to conduct so-called "mismatch analyses" (e.g., response surface analyses 147 ) to explore discrepancies between expectations and actual experiences during childbirth and their effect on the subjective birth experience of parents. Growth curve analysis can be conducted to investigate changes in birth experiences from T2 to T4. Growth curve analyses can also be performed with predictor variables, for example, to evaluate if obstetric violence/mistreatment during childbirth predicts the course of the subjective birth experience. Dyadic analyses, such as dyadic response surface analysis 148 , can be used to analyse the interactions between two or more variables within dyadic ([expectant] mother/birthing parent and partner) data (e.g., whether a mismatch of expectations between mothers/birthing parents and partners predict their CB-PTSD symptoms 149 ). Actor-Partner Interdependence Models (APIM) 150 can be used to investigate partner effects among parents (e.g., the subjective birth experience of one parent as a predictor of the other parent’s bonding with the child 12 ).

All analyses will be performed controlling for potential covariates (e.g., socioeconomic status, being [expectant] first-time parent, gestational week at T1). If a statistical method only permits dichotomous variables, variables will be dichotomised for the analyses. However, whenever possible it is preferred to use continuous variables to avoid loss of information. A significance level of p < 0.05 with a 95% confidence interval and two tailed tests will be used for all analyses. When computing structural equation models, model fit will be evaluated using model fit indices such as the comparative fit index (CFI) 151 , the root mean squared error of approximation (RMSEA) 152 , or the square root mean residual (SRMR) 153 . The specific indices are selected depending on, for example, the complexity of the model and the sample size, with the aim of selecting indices that are best suited for assessing the adequacy of the model.

When computing growth curve analyses, latent variables will be used wherever possible. When adding predictors to the growth curve models, in a first step, an unconditional model not including the predictors will be computed to analyse the overall pattern of the outcome variable over time. In a second step, a conditional model including all predictors of the trajectory of the outcome variable will be computed. The handling of the time scores for each measurement time point of the outcome will be decided separately for each research question. For the slope, most research questions will test both linear as well as quadratic latent growth factors to see which fits the data best.

Missing values in single items of questionnaires will be replaced by the participants’ mean value if less than 20% of the items in the respective questionnaire are missing, unless otherwise described. Whenever possible, Full Information Maximum Likelihood estimation will be used to estimate missing scores in variables, as this technique is considered the most adequate approach for dealing with missing data 154 . If this technique is not applicable for the chosen computation, listwise deletion will be used.

With data collection planned for a follow-up period of at least 24 months and data analysis extending beyond this time frame, new statistical methods may become available that motivate reprocessing or re-analysing of the data to answer new research questions.

#### Qualitative data analysis

The data collected within the qualitative interviews of both RESPECT _PARENTS-TALK_ and RESPECT _STAFF_ (interview transcripts) will be analysed using reflexive thematic analysis (rTA) by Braun & Clarke 155 156 , which consists of the following six steps: 1) familiarisation with the data, 2) coding, 3) searching for themes, 4) reviewing themes, 5) defining and naming themes, 6) and writing up.

This approach was mainly designed to identify patterns of meaning across a data set and is currently widely used in qualitative health research. The reflexive approach puts special focus on the role of the researchers and their own attitudes and beliefs, creating robust, transparent, and unbiased results. Reflexivity emphasises the subjectivity of results and improves the quality of qualitative data analysis. As a result of rTA, the aim is to identify themes across the datasets that best describe the thought processes, attitudes, and beliefs of the participants in order to develop suggestions for improvements for obstetric care and higher levels of person-centredness.

#### Considerations regarding sample size and power calculations

With regard to the quantitative main study, an a-priori power analysis for multiple linear regression analyses was performed using G*Power 3.1.9.2 to determine whether the planned sample size is sufficient for the key research questions. Power analysis for multiple linear regression analyses was chosen, because they represent a significant portion of the analyses and will address the central research question of which factors predict parents’ birth experiences. The results of the power analysis indicated that a sample size of at least n = 647 expectant mothers/birthing parents and n = 647 partners is required to detect small effects (Cohen’s ƒ ^2^ = 0.02) with 80% power and alpha = 0.05 within a multiple linear regression analysis including five predictors. As our targeted sample size exceeds these minimum requirements, it can be assumed that the planned analyses can be conducted. However, to proactively mitigate potential attrition and to conduct more advanced analyses (e.g., dyadic analyses), it may be beneficial to include more than n = 647 expectant mothers/birthing parents and partners. Therefore, we computed the maximum number of expectant mothers/birthing parents and partners that could be recruited within a recruitment period of at least 16 months. This computation was based on the retention rates from previous studies in the same hospitals and a freestanding birth centre 9 86 , supplemented by the assumption that study nurses are likely to exceed these retention rates through their personal approach. This led to the aim of recruiting n = 1680 expectant mothers/birthing parents and n = 880 partners, which should be sufficient even for advanced analyses like dyadic data analysis, because it exceeds the sample sizes of most previous studies, which reported sufficient statistical power for similar statistical designs, and accounts for participant attrition.

Within qualitative research, theoretical saturation is a common concept used to determine the sample size that is large enough to fully explore the topic 157 158 , meaning that further data collection would not provide additional information on the phenomenon of interest. Generally, saturation is suggested to be reached by including around 12 participants of a homogeneous group 159 . However, more recent approaches recommend approximating the number of participants needed for qualitative research based on the “information power” of a particular data set 150 , taking into account the research aims, the specificity of the sample, the theoretical basis, the quality of data already collected, and the analytical approach. Based on this concept, which is also recommended by Braun and Clarke 160 for projects using reflexive thematic analysis, sample sizes can hardly be estimated in advance. However, our quite specific research questions combined with rather homogenous participant groups, as well as the recommendation of around 12 participants according to saturation considerations led to the aim of recruiting 10–15 participants for each target group within RESPECT _PARENTS-TALK_ and RESPECT _STAFF_ . Thus, within RESPECT _PARENTS-TALK_ , it is planned to conduct 10–15 interviews with mothers/birthing parents and 10–15 interviews with partners. For RESPECT _STAFF_ , 10–15 interviews are planned with members of each professional group (i.e., midwives and obstetricians). The planned sample sizes for both RESPECT _PARENTS-TALK_ and RESPECT _STAFF_ will be adjusted continuously in the process of data collection with respect to the quality and depth of the interviews already conducted. Furthermore, for RESPECT _STAFF_ , we will also take into account the responses to the short questionnaire on the registration website in order to achieve diversity in the initial attitudes of participating obstetric health care staff towards PCC in obstetrics.

## First Results

In the following, preliminary results regarding the sample of the main study RESPECT _PARENTS_ are presented.

### Sample and interim flow chart of RESPECT _PARENTS_

**Fig. 3:**
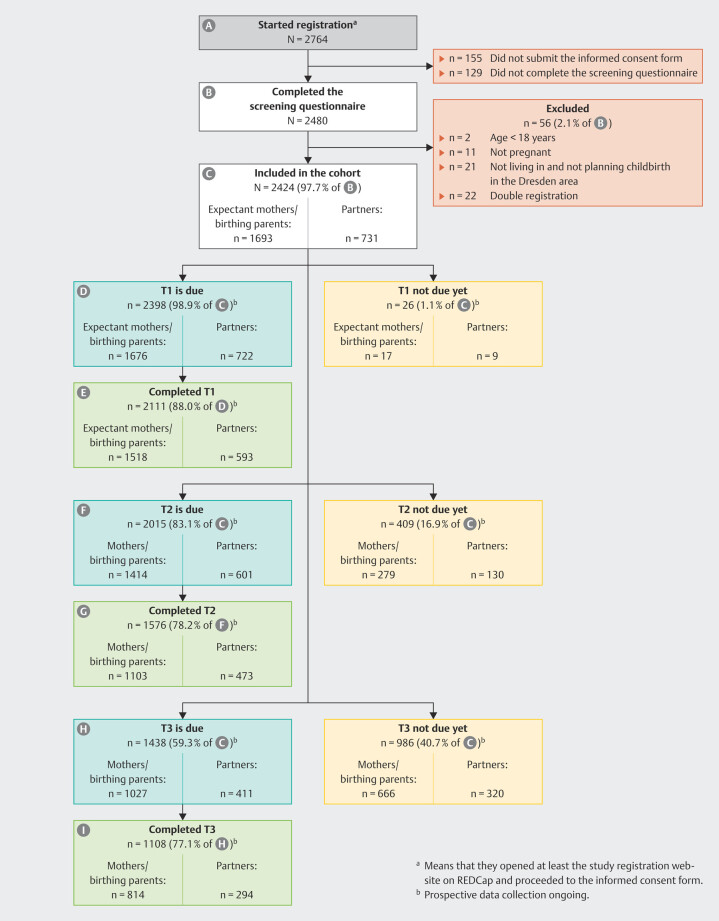
Interim flowchart of parents participating in RESPECT _PARENTS_ .

The interim flow chart of RESPECT _PARENTS_ is presented in Fig. 3 . During the recruitment period of approximately 21 months, a total of N = 2764 parents initially started the registration process. Of the N = 2480 parents who completed the screening questionnaire, a total of N = 2424 parents (97.7%) were eligible for study participation, resulting in a final sample of n = 1693 expectant mothers/birthing parents (69.8%) and n = 731 partners (30.2%), including n = 14 female partners (0.6%). For several reasons (see Fig. 3 ), n = 56 parents were excluded after completing the screening questionnaire.

At study registration, n = 1274 participants (52.6%) joined the study as a couple, whereas n = 1056 expectant mothers/birthing parents (43.6%) and n = 94 partners (3.9%) joined without the other expectant parent.

The vast majority of participants (n = 1544; 63.7%) was recruited at the University Hospital Dresden, a Perinatal Centre Level 1. Additionally, n = 560 participants (23.1%) were recruited through other maternity hospitals in Dresden and n = 37 (1.5%) through a freestanding birth centre led by midwives. Of the additional recruitment channels, advertising through social media was the one that yielded the largest number of participants (n = 114; 4.7%). The table presenting the recruitment numbers per place can be found in Supplementary **Table S2** .

Until January 2, 2025 (data export date), the first online questionnaire (T1) had been activated for n = 2398 participants whose pregnancy had reached the 24 ^th^ gestational week or beyond. Of those, n = 2111 participants (n = 1518 expectant mothers/birthing parents, and n = 593 partners) completed T1, providing baseline sociodemographic and health-related characteristics during their pregnancy. This corresponds to a preliminary retention rate of 88.0% at T1. However, as T1 was still active for some participants whose children had not been born until the date of data extraction for this study protocol, the retention rate is expected to further increase until data collection for T1 is completed. Expectant mothers/birthing parents were significantly more likely to complete the T1 assessment compared to partners (χ² = 33.13, p < 0.001). Parental age and gestational week at study registration were not associated with T1 completion. With regard to the T2 assessment eight weeks after the anticipated childbirth, n = 2015 participants were already contacted by the study team to schedule the quantitative telephone interview. Of those, n = 1576 participants were interviewed, resulting in a preliminary retention rate of 78.2% at T2. As some of the participants still have the chance to participate in the interview, the retention rate again is likely to be higher at the end of data collection for T2. A total of eight (expectant) mothers and three partners (i.e., three couples and five mothers participating alone) reported either the experience of miscarriage during early pregnancy or stillbirth of their child, therefore their study participation ended prematurely.

Data collection for T3 (six months after childbirth) started in October 2023. T4 assessment, 24 months after childbirth, is expected to begin in April 2025.

### Sample of RESPECT _PARENTS-TALK_ and RESPECT _STAFF_

With regard to the qualitative sub-study RESPECT _PARENTS-TALK_ , n = 14 mothers and n = 14 partners have been interviewed. For RESPECT _STAFF_ , n = 27 interviews with obstetric health care staff (n = 14 midwives and n = 13 obstetricians) have been conducted.

### Sociodemographic and pregnancy-related characteristics

Table 2 displays preliminary sociodemographic characteristics of the RESPECT _PARENTS_ sample. The mean age was 32.0 years (SD = 5.0; Range 18–49) for expectant mothers/birthing parents and 34.1 years (SD = 5.6; Range 20–59) for partners. The majority of participants (90.3%) was born in Germany and reported German to be their mother tongue (91.1%). This suggests that the current sample may contain a slightly higher proportion of people with German citizenship than the Dresden population (87.8%) 161 . Of all participants, 4.4% would describe themselves as a member of a population group that is discriminated against in Germany, because of, e.g., nationality, language, or sex, and another 4.7% were not sure about this.

**Table:** **Table 2** Sociodemographic and pregnancy-related characteristics of expectant mothers/birthing parents and partners assessed at T1.

	All participants		Expectant mothers/birthing parents only		Partners only	
	(n = 2111)		(n = 1518)		(n = 593)	
	n ^a^	% ^b^	n ^a^	% ^b^	n ^a^	% ^b^
**Sex**						
Female	1521	72.5	1510	100.0	11	1.9
Male	577	27.5	0	0.0	577	98.1
**Gender identity**						
Female	1503	72.2	1492	99.7	11	1.9
Male	573	27.5	1	0.1	572	97.6
Agender	1	0.1	1	0.1	0	0
Refused to be categorised by gender	4	0.2	1	0.1	3	0.5
**Relationship status**						
In a permanent relationship	2065	98.4	1478	97.9	587	99.7
Not in a permanent relationship	34	1.6	32	2.1	2	0.3
**Marital status**						
Married/registered same-sex partnership	1158	55.0	823	54.4	335	56.6
Never married	886	42.1	644	42.6	242	40.9
Separated/widowed/divorced	61	2.9	46	3.0	15	2.5
**Educational qualification**						
No degree (yet)	7	0.3	4	0.3	3	0.5
Lower secondary education level 2	71	3.4	54	3.6	17	2.9
Secondary school certificate	507	24.2	377	25.1	130	22.0
Subject-related or higher education entrance qualification (A-level)	1427	68.1	1015	67.5	412	69.8
Other school-leaving qualification (e.g. obtained abroad)	82	3.9	54	3.6	28	4.7
**Professional qualification**						
No qualification (yet)	103	4.9	81	5.4	22	3.7
Occupational apprenticeship	699	33.4	534	35.5	165	28.0
Master of crafts	142	6.8	83	5.5	59	10.0
Degree from a technical or an engineering college	104	5.0	68	4.5	36	6.1
University degree	1007	48.1	712	47.3	295	50.1
Other training qualification (e.g. obtained abroad)	38	1.8	26	1.7	12	2.0
**Occupational status**						
Full-time employed	1386	66.3	896	59.7	490	82.9
Part-time employed	522	25.0	454	30.3	68	11.5
Marginally employed	44	2.1	30	2.0	14	2.4
None of the above	139	6.6	120	8.0	19	3.2
**Country of birth**						
Germany	1901	90.3	1367	90.4	534	90.2
Other	204	9.7	146	9.6	58	9.8
**Mother tongue**						
German ^c^	1914	91.1	1376	91.1	538	91.0
Other	188	8.9	135	8.9	53	9.0
** Member of a discriminated population group (self-attribution) ^d^**						
No	1899	90.9	1359	90.7	540	91.4
Yes	92	4.4	62	4.1	30	5.1
Don’t know	98	4.7	77	5.1	21	3.6
**Number of children**						
0	1269	61.0	855	57.3	414	70.4
1	612	29.4	475	31.8	137	23.3
2	159	7.6	130	8.7	29	4.9
3	29	1.4	23	1.5	6	1.0
4 or more	12	0.6	10	0.7	2	0.4
**Number of children expected**						
One child	2033	96.3	1462	96.3	571	96.3
Twins	78	3.7	56	3.7	22	3.7
	**M (SD)**	**Range**	**M (SD)**	**Range**	**M (SD)**	**Range**
Age in years	32.6 (5.4)	18–59	32.0 (5.0)	18–49	34.1 (5.6)	20–59
Gestational week at T1 ^e^	32.8 (4.8)	24–42	32.8 (4.8)	24–42	32.8 (4.9)	25–42
Note: ^a^ n varies slightly due to missing data of some participants. ^b^ Valid percent (due to rounded percentages, percentages add up to 99.9% or 100.1% in some cases). ^c^ Including German only and German plus another language. ^d^ The following reasons for discrimination were specified in the overall sample: n = 18 skin colour, n = 38 nationality, n = 13 ethnic group, n = 18 language, n = 15 religion, n = 24 sex, n = 23 sexual orientation, n = 2 handicap/chronic disease, n = 2 age, and n = 7 other reasons. ^e^ T1 assessment during late pregnancy.						

The majority (68.1%) reported a subject-related or higher education entrance qualification (A‑level) and 48.1% held a university degree, indicating a rather high educational and professional level compared to the general German population 162 and the population of Dresden 163 . Almost all participants (98.4%) were currently in a permanent relationship and slightly more than half (55.0%) were married.

On average, expectant parents registered for study participation in gestational week 31.5 (SD = 6.4; Range = 5–42) and completed the T1 assessment in gestational week 32.8 (SD = 4.8; Range = 24–42). Twin pregnancies were reported by 3.7% of the expectant parents. Higher-order pregnancies with three or more fetuses were not reported. About half (57.3%) of the expectant mothers/birthing parents were expecting their first child, while this was true for two thirds of the partners (70.4%). Of expectant mothers/birthing parents expecting their first child, the average age was 30.9 years (SD = 5.0; Range 18–49), which is comparable to the average in Saxony (29.5 years) and Germany (30.3 years) 164 . Partners in our sample were 33.0 years (SD = 5.2, Range 20–48) old on average when expecting their first child, which is also comparable to the German average (33.2 years) 165 .

### Birth-related characteristics

Birth-related characteristics assessed at T2 are presented in Table 3 . T2 data were available from n = 1576 participants (n = 1103 mothers/birthing parents; n = 473 partners) whose children were born between April 2023 and the mid of November 2024. The interviews took place on average 9.7 weeks after the actual date of birth (SD = 2.7; Range = 5–23 weeks). Subsequently, data from n = 1193 families in which at least one parent (mother/birthing parent and/or partner) participated in the T2 interview are reported. In case both parents participated and they provided different information on objective birth-related outcomes (e.g., place of birth or mode of birth), we report the data provided by the mother/birthing parent, as most of the objective information collected relates more directly to the mother/birthing parent and it can be assumed that their information is more accurate.

**Table:** **Table 3** Birth-related characteristics as reported either by mothers/birthing parents, partners, or both at T2.

	Families		Mothers/birthing parents		Partners	
	(n = 1193)		(n = 1103)		(n = 473)	
	n ^a^	% ^b^	n ^a^	% ^b^	n ^a^	% ^b^
**Number of children born**						
One child	1158	97.1	1070	97.0	461	97.5
Twins	35	2.9	33	3.0	12	2.5
** Sex of child ^c^**						
Female	584	48.0	538	48.0	238	49.1
Male	632	52.0	584	52.0	247	50.9
**Place of birth**						
Maternity hospital	1157	97.1	1068	96.9	457	96.6
Freestanding birth centre	16	1.3	15	1.4	8	1.7
Home birth	18	1.5	18	1.6	8	1.7
On the way	1	0.1	1	0.1	0	0.0
** Level of perinatal care of the maternity hospital ^d^**						
Perinatal Centre Level 1	778	67.2	724	67.8	288	63.0
Perinatal Centre Level 2	104	9.0	94	8.8	41	9.0
Perinatal focus (Level 3)	200	17.3	180	16.9	95	20.8
Birth clinic (Level 4)	173	6.3	67	6.3	33	7.2
Not known	2	0.2	2	0.2	0	0.0
**Mode of birth**						
Vaginal	815	68.4	755	68.6	322	68.2
Vaginal-operative ^e^	65	5.5	61	5.5	21	4.4
Elective/planned caesarean section	130	10.9	120	10.9	52	11.0
Unplanned caesarean section	149	12.5	138	12.5	61	12.9
Emergency caesarean section	32	2.7	27	2.5	16	3.4
**Induction of labour**						
No	788	66.1	740	67.2	269	56.9
Yes	399	33.5	358	32.5	197	41.6
Unclear	5	0.4	3	0.3	7	1.5
** Preterm birth ^f^**						
No	1089	91.3	1007	91.3	441	93.2
Yes	104	8.7	96	8.7	32	6.8
**Attending birth as accompanying person**						
No	n.a.	n.a.	n.a.	n.a.	9	1.9
Yes	n.a.	n.a.	n.a.	n.a.	464	98.1
	**M (SD)**	**Range**	**M (SD)**	**Range**	**M (SD)**	**Range**
Gestational week at birth	39.8 (2.0)	27–43	39.8 (1.9)	27–43	40.0 (1.9)	28–43
Time since birth at the time of the interview ^g^	9.7 (2.7)	5–23	9.7 (2.7)	6–23	9.6 (2.7)	5–20
Note: ^a^ n varies slightly due to missing data of some participants. ^b^ Valid percent (due to rounded percentages, percentages add up to 99.9% or 100.1% in some cases). ^c^ Missing information for n = 12 children out of 1228 children born. ^d^ Only applicable to births in a maternity hospital (n = 1157). ^e^ With forceps or vacuum extraction. ^f^ Childbirth before completing the 37 ^th^ gestational week. ^g^ In weeks.						

The 1193 interviewed families (n = 383 couples; n = 720 mothers/birthing parents only; n = 90 partners only) reported the birth of 1228 children (2.9% multiple births). The ratio of female (48.0%) to male children (52.0%) was nearly balanced and comparable to the statistics reported for all childbirths in Dresden in 2023 (47.6% female) 166 and 2024 (48.8% female) 167 . With the exception of n = 18 home births (1.5%), n = 16 childbirths in a freestanding birth centre (1.3%), and n = 1 child born on the way, all children (97.1%) were born in a maternity hospital and most of them (67.2%) in a Perinatal Centre Level 1. On average, the children were born at 39.8 gestational weeks (SD = 2.0; Range = 27–43) with n = 104 families (8.7%) reporting a preterm birth (defined as childbirth before completing the 37 ^th^ gestational week). This rate of preterm births is slightly higher compared to the German average of 7.9% 168 . Almost three quarters of families (n = 880; 73.9%) reported a vaginal birth, including 5.5% who had a vaginal-operative birth. The percentage of caesarean sections (26.1%; n = 311) is lower compared to the German average with 32.6% and representative for Saxony with 25.6% 169 . An induction of labour was reported by n = 399 (33.5%) of the interviewed families. The majority of mothers/birthing parents (93.4%) were accompanied by their partner during childbirth and 98.1% of partners reported that they were present at birth.

## Discussion

The interdisciplinary research project RESPECT is one of the first to systematically investigate the subjective birth experience of both parents including a variety of associated factors before, during, and after childbirth. Moreover, RESPECT will provide unique systematic data on the types and frequency of mistreatment during childbirth in Germany, as well as its consequences for the subjective birth experience and postpartum (mental) health outcomes. It further provides detailed insights into the perspectives of parents and obstetric health care staff on PCC during childbirth, allowing the perspectives of both target groups to be compared and recommendations for action to be derived.

In sum, RESPECT has several major strengths. By adopting a prospective longitudinal design within the quantitative main study RESPECT _PARENTS_ (i.e., presumably four assessment points covering a period from late pregnancy up to 24 months after childbirth), it is possible to account for multiple factors in the perinatal period and to integrate them into advanced analyses. With the qualitative sub-study (RESPECT _PARENTS-TALK_ ) and the additional qualitative study branch (RESPECT _STAFF_ ), attitudes and beliefs of both parents and obstetric health care staff regarding PCC and the role of obstetric health care staff during childbirth can be explored in detail. This mixed-methods approach combines quantitative data, primarily assessed using well-established instruments, with qualitative data from semi-structured, guideline-based interviews, providing a comprehensive picture of the subjective birth experience and a deeper insight into the underlying meaning of the quantitative data. Altogether, this methodology allows for answering research questions that are both longitudinal and cross-sectional in nature. Importantly, by involving (expectant) mothers/birthing parents, partners, and obstetric health care staff as target groups, RESPECT captures multiple perspectives. Furthermore, the RESPECT Advisory Board was actively involved from the beginning of the project, providing invaluable input to the study (e.g., by discussing methodological questions and project milestones) and significantly strengthening the quality of the research output. The involvement of Mother Hood e. V. in addition to perinatal researchers and practicing obstetric health care staff ensured that the parents’ perspective was also considered in the planning and implementation of RESPECT. This supports the idea of participatory research in line with international expert consensus recommendations to involve relevant target groups in research on birth-related issues in order to guarantee the relevance and legitimacy of research outcomes 170 . Lastly, the large community sample of the quantitative main study RESPECT _PARENTS_ including N = 2424 participants in total, provides an opportunity to apply advanced statistical methods such as multi-level modeling. A variety of multifaceted recruitment strategies was implemented with the intention of approaching all expectant parents both German- and English-speaking in the Dresden area. These strategies included collaboration with all of Dresden’s maternity hospitals, which offer different levels of perinatal care, a freestanding birth centre, and other institutions (e.g., pregnancy counselling services and a regional network supporting men and fathers), as well as gynaecologists’, midwives’ and pediatricians’ practices that support the RESPECT project. By combining these dominant recruitment strategies with additional indirect strategies such as social media, we aimed for a cohort that is diverse in terms of age, socioeconomic status, migration background, and planned birth setting. Due to the primary recruitment at the University Hospital Dresden including the consultation for high-risk pregnancies, there is a chance that high-risk pregnancies may be overrepresented in the sample, which needs to be checked in subsequent analyses and discussed accordingly.

Descriptive analyses of the sample show that the ratio of primiparous to multiparous expectant mothers/birthing parents is nearly balanced. This makes the cohort particularly valuable, as parity has been identified as a significant covariate in some studies 39 171 . In contrast, the proportion of first-time parents is higher among the partners in our sample, with almost two thirds expecting their first child. This was expected as partners are more likely to accompany their childbearing partner to information events, gynaecological visits, and antenatal classes if they are expecting a child for the first time, and thus are more likely to be approached for participation in RESPECT _PARENTS_ . In subsequent pregnancies, accompanying partners may be less common, for example, if partners stay home with older children during these appointments. Overall, the number of partners in our sample is lower than expected. This is consistent with previous research indicating that male individuals are less likely to engage in epidemiological studies and partners are more likely to be male 172 173 174 . The response rate of partners was also lower than that of expectant mothers/birthing parents in the Dresden Study on Parenting, Work, and Mental Health (DREAM) 9 , another large epidemiological study in the field of perinatal (mental) health conducted in the Dresden area and targeting couples. As RESPECT examines the subjective birth experience, partners may be less inclined to perceive the study’s focus as relevant to them compared to expectant mothers/birthing parents. In support of this, research shows that when asked about their birth experience, partners often talk about the mothers’/birthing parents’ experienced pain and needs during childbirth, suggesting that they may not feel their own experience to be equally relevant 50 175 . It is also common for partners to be seen as companions during pregnancy and childbirth, rather than as having needs of their own. In general, it is more difficult to approach partners in the perinatal setting than it is to approach expectant mothers/birthing parents. Despite ongoing efforts within RESPECT _PARENTS_ to address them directly, using tailored leaflets and recruiting at events specifically offered to expectant fathers, the lower response rate from partners seems to be persistent. Overall, the monthly recruitment rate for RESPECT _PARENTS_ was lower than expected for both expectant mothers/birthing parents and partners. This might partly be explained by the declining birth rates in Germany, which decreased by 7% in 2022 and by a further 6% in 2023 176 , with the decline in birth rates continuing in 2024 177 . Nevertheless, the final number of participants included in the cohort exceeds the needed sample size calculated in the a-priori power calculation. Therefore, the sample is expected to have sufficient power to answer the research questions of RESPECT and even exceeds the sample sizes of most previous studies, especially for partners, thereby retaining the ability to perform advanced analyses.

RESPECT _PARENTS_ aimed for a community sample of (expectant) partners that is representative of the population of Dresden in terms of sociodemographic variables by approaching almost all expectant parents in the Dresden area. However, representativeness in terms of certain variables, such as socioeconomic status, is difficult to achieve. Our sample is mainly composed of well-educated individuals with a higher professional qualification and socioeconomic status. This bias was also found in another recent study among mothers in the Dresden area 86 and may be due to the fact that individuals with higher socioeconomic status are generally more likely to participate in research 173 174 because they may face fewer barriers (e.g., health literacy), may be more likely to feel that the research is relevant to them, and may be more aware of the importance and benefits of participating in such studies. Unfortunately, self-selection of participants according to, e.g., sociodemographic variables is a common and well-known issue in epidemiological studies. Nevertheless, studies explicitly investigating the impact of self-selection with respect to initial and follow-up participation in birth and pregnancy studies have found that while self-selection can influence estimates of the prevalence of certain variables (e.g. educational level), it appears to have little or no effect on estimates of the associations between variables (e.g. the association between educational level and gestational age or infant birth weight 178 ; see also 173 179 180 181 ). Due to limited financial and personal resources, it was not possible to facilitate study participation in other languages than German or English, which can be associated with the low percentage of migrants, that is, however, close to be representative for Dresden 161 . The findings regarding the representativeness of the sample highlight the need for caution in generalising the findings beyond the demographics of the RESPECT _PARENTS_ study population. However, the insights gained from the study will still be valuable within the context of the research questions, especially regarding the associations between variables, and sociodemographic variables will be controlled for within statistical analyses, which will mitigate their impact on the results and improve generalisability.

Regarding first birth-related characteristics of the sample, the majority of families (73.9%) reported a vaginal birth mode, with the low percentage of caesarean sections being representative of the region 169 . There is a slightly higher preterm birth rate in our sample compared to the German average (8.7% vs. 7.9%) 168 . This might be due to the high percentage of births in a Level 1 Perinatal Centre, which provides the highest quality of care for babies born prematurely. The ratio of twin births in our interim sample (2.9%) is slightly higher than in the statistics reported for all childbirths in Dresden in 2023 (1.6%) 166 and 2024 (1.8%) 167 , which again may be explained by a most likely overrepresentation of births in a Level 1 Perinatal Centre, for which higher ratios of twin births are reported (e.g., 182 ). As expected, the rate of births in out-of-hospital settings (i.e., home births or births in freestanding birth centres; 2.8%) was low, but higher compared to the rate of 2.0% reported for Germany in 2023 183 . Despite the small number of cases in the sample, it is essential to include these birth settings as well in order to reflect the full spectrum of birth experiences. Also, including them helps identify factors that may contribute to the anticipated more positive outcomes in these contexts, which can inform general improvements in childbirth care.

Overall, the data generated within RESPECT will provide meaningful insights into both parents’ subjective birth experiences, their predictors, and their (mental) health-related outcomes, including barriers and facilitators regarding the provision of PCC from the perspective of obstetric health care staff. As the subjective birth experience can be of great importance to families, it is essential to identify factors and measures that can positively influence the parental subjective birth experience. In order to implement person-centred approaches to obstetric health care as a potentially decisive factor, the perspective of obstetric health care staff is crucial. While PCC approaches must represent the perspective of parents, its implementation is the responsibility of the obstetric health care staff. The results of RESPECT _STAFF_ can therefore be used as a basis for developing solutions that meet the needs and realities of obstetric health care staff and facilitate the implementation of PCC in daily obstetric practice. In addition, the potential for improving the work situation and satisfaction of obstetric health care staff can be identified and used as a starting point for further action.

From an economic or societal perspective, the experiences parents have during childbirth – including mistreatment and disrespect on the one hand, and PCC on the other – reverberate far beyond the immediate impact on mothers/birthing parents, partners, and their families. For instance, a negative or traumatic birth experience may result in postpartum mental health disorders (such as CB-PTSD or postpartum depression), necessitating pharmacological and/or psychological treatment that is costly to the health care system 184 185 186 . Furthermore, negative subjective childbirth experiences can lead to the decision not to have any more children, thus affecting the country’s fertility rate 28 . Given its implications for population development, health care utilisation and the overall public health impact, the subjective birth experience and the quality of childbirth care is a topical field of research, both on a national and international level.

The RESPECT project forms the basis for several publications that will increase our knowledge of the interrelations between individual, birth-, and care-related factors in the context of the subjective birth experience and childbirth-related (mental) health outcomes, with a special focus on the role of obstetric health care staff in parents’ birth experience. The results of RESPECT are intended to inform politicians, health policy-makers, and health care institutions to identify strategies and possibly design pilot projects to further improve the quality of obstetric health care and associated services in Germany in order to facilitate a positive subjective birth experience in accordance with the latest WHO recommendations 45 . A workshop for experts and representatives of relevant professional groups and the scientific community, and several conference presentations are planned to disseminate the results and make them available to the professional public. In addition to answering the key research questions of RESPECT, validation studies in a German population are planned for instruments used in the main study RESPECT _PARENTS_ , e.g. for the WP-RMC 118 , which is used to assess PCC in obstetrics. Furthermore, data from RESPECT can be compared with two other large longitudinal (DREAM 9 ) and cross-sectional (INVITE 86 ) epidemiological studies in the field of perinatal (mental) health conducted in the Dresden area. Particularly the DREAM study has similar assessment points (T1: late pregnancy, T2: 8 weeks postpartum, T4: 24 months postpartum) and uses similar instruments (e.g., the EPDS for pre- and postpartum depression, or the PBQ for postpartum parent-child bonding) as RESPECT. Thus, data from both projects may be combined in order to answer questions even more reliably on the basis of a larger sample size. This is especially valuable in terms of analyses with small subgroups, e.g., participants with a migration background or with full-syndrome CB-PTSD. Likewise, the recently completed INVITE study with a single assessment point 3–4 months after the birth utilised some of the same instruments for which data can be combined.

Ultimately, the findings of the RESPECT project will contribute to the development of measures in line with the WHO recommendations on “intrapartum care for a positive childbirth experience” 45 and the German national health goal “Health around childbirth”, an initiative of the German government aimed at strengthening the well-being of mother and child in this vulnerable phase of life 187 . Both explicitly emphasise the subjective well-being in addition to the somatic health of the mother/birthing parent and the newborn. Although global and national public health goals do not include partners, enhancing the subjective birth experience of both parents is crucial for fostering strong and healthy families in the perinatal period. Consequently, recommendations for actions to further improve the quality of childbirth care with special focus on the subjective birth experience should include both parents. At the same time, measures that reflect the perspectives and needs of obstetric health care staff are needed in order to promote the implementation of PCC in everyday clinical practice.

## Supplementary Material

- **Table S1.** Pre-defined screening criteria for RESPECT _PARENTS-TALK_ .
- **Table S2.** Places of recruitment of the main study RESPECT _PARENTS_ .

## Ethics Statement

The studies involving human participants were reviewed and approved by the Ethics Committee of the Technische Universität Dresden (No: SR-EK-331072022).

## Footnotes

1 including female, trans, inter, agender, and non-binary birthing parents.

2 including biological fathers as well as other male, female, trans, inter, agender, and non-binary non-birthing parents.

3 In Germany, maternity hospitals are categorised into four care levels (Perinatal Centre Level 1, Perinatal Centre Level 2, Perinatal focus [Level 3], Birth clinic [Level 4]) according to the “Quality Guideline of Care Delivery of Preterm and Mature Infants” (“Qualitätssicherungs-Richtlinie Früh- und Reifgeborene/QFR-RL”; Gemeinsamer Bundesausschuss, 2024). Each level reflects the maternity hospital’s capabilities and resources to handle various complexities of pregnancy and childbirth (e.g., low-risk and high-risk pregnancies, or preterm births).

## Supplementary Material

Supplementary Material Zusatzmaterial
