# Supplementary material for: Subjective Birth Experience and Person-Centred Care in Obstetrics: Study Protocol of the Prospective Mixed-Methods Research Project RESPECT
Source: Geburtshilfe Frauenheilkd. 2025 Jun 2;85(11):1169–94. doi: 10.1055/a-2551-3705 (PMC12591835; doi:10.1055/a-2551-3705)
Supplement: Supplementary file 1 — Supplementary Material [file 10-1055-a-2551-3705_25513878.pdf]

Table S1 Pre-defined screening criteria for RESPECT$_{\text{PARENTS-TALK}}$.

Main criteria	Secondary criteria	Additional criteria
Perception of interactions with obstetric health care staff	Number of previous births	Country of birth/mother tongue
Subjective birth experience	Birth expectations	Age
Mode of birth	Wishes regarding interventions and match with interventions performed	Educational level
Number of interventions	Place of birth (hospital, birth centre, or home birth)	Level of perinatal care of maternity hospital [a]
	Birth duration	Pregnancy complications
	Birth complications	Preterm birth
	Desired mode of birth and match with actual mode of birth	Transferal during birth [b]
	Experiences of discrimination during birth	Presence of accompanying person(s)
		Interventions without consent
		Childbirth-related posttraumatic stress symptoms

Note. [a] In Germany, maternity hospitals are categorised into four care levels (Perinatal Centre Level 1, Perinatal Centre Level 2, Perinatal focus [Level 3], Birth clinic [Level 4]) according to the “Quality Guideline of Care Delivery of Preterm and Mature Infants” ["Qualitätssicherungs-Richtlinie Früh- und Reifgeborene/QFR-RL”; 88]. Each level reflects the maternity hospital's capabilities and resources to handle various complexities of pregnancy and childbirth (e.g., low-risk and high-risk pregnancies, or preterm births). [b] Change of place of birth, e.g., to gain access to a higher level of care.

Thieme

Table S2 Places of recruitment of the main study $RESPECT_{PARENTS}$.

	**All recruited participants (*N* = 2,424)**		**Expectant mothers/ birthing parents only (*n* = 1,693)**		**Partners only (*n* = 731)**	
	n	%	*n*	%	*n*	%
Maternity hospital (all levels [a])	2,104	86.8	1,466	86.6	638	87.3
Perinatal Centre Level 1 [b]	1,544	73.4	1,104	75.4	440	69.0
Perinatal Centre Level 2 [c]	165	7.8	108	7.3	57	8.9
Perinatal focus (Level 3) [d]	302	14.4	197	13.4	105	16.5
Birth clinic (Level 4) [e, f]	93	4.4	57	3.9	36	5.6
Freestanding birth centre	37	1.5	22	1.3	15	2.1
Gynaecological practice	26	1.1	22	1.3	4	0.5
Midwife practice	20	0.8	17	1.0	3	0.4
Other parents, friends, or relatives (including partner)	51	2.1	23	1.4	28	3.8
Social media and websites	114	4.7	93	5.5	21	2.9
Baby fairs	42	1.7	28	1.7	14	1.9
Kindergarten	7	0.3	7	0.4	0	0.0
Other places	19	0.8	11	0.6	8	1.1
Unknown places	4	0.2	4	0.2	0	0.0

Note. [a] Level of maternity hospitals according to the "Quality Guideline of Care Delivery of Preterm and Mature Infants" ["Qualitätssicherungs-Richtlinie Früh- und Reifgeborene/QFR-RL"; 88]. [b] Perinatal Centre Level 1 are advanced facilities equipped to handle the highest-risk pregnancies and births (including extremely premature babies) with extensive neonatal intensive care units and a full range of multi-professional medical specialists available 24/7. [c] Perinatal Centre Level 2 manage high-risk pregnancies with slightly less complexity than Level 1, featuring neonatal care and essential specialist support. [d] Maternity hospitals with a perinatal focus (Level 3) cater to low to moderate-risk pregnancies, providing standard delivery and neonatal care, referring higher-risk cases to higher-level centres. [e] Birth clinics (Level 4) focus on normal pregnancies and births from 36 week of gestation onward, offering standard obstetric care and transferring high-risk cases to higher-level facilities. [f] Due to organisational and personnel reasons, recruitment at the level 4 birth clinic started one year later than at the other maternity hospitals, which contributed to the smaller number of participants recruited at this place.
